# Identification and functional analysis of αB-crystallins in *Pteromalus puparum*

**DOI:** 10.3389/fphys.2023.1214835

**Published:** 2023-07-14

**Authors:** Shuxing Lao, Shijiao Xiong, Qi Fang, Gongyin Ye

**Affiliations:** State Key Laboratory of Rice Biology and Breeding, Institute of Insect Sciences, Zhejiang University, Hangzhou, China

**Keywords:** parasitoid wasp, HSP, CRYAB, heat stress, lifespan, RNAi

## Abstract

Heat shock proteins, including αB-crystallins (CRYAB), are pivotal in cellular defense mechanisms and stress response. This study presents a comprehensive investigation of heat shock proteins (HSPs), with a specific focus on the CRYAB family, within the genome of *Pteromalus puparum*. The analysis encompasses the identification of these proteins, exploration of their phylogenetic relationships, examination of conserved domains, and evaluation of their response to high temperature conditions. A total of 46 HSPs were identified in the *P. puparum* genome, and the differential expression of mRNA at 35°C and 25°C drew attention to five genes belonging to the CRYAB family, namely, *PpCRYAB-1* to *PpCRYAB-5*. The conservation level of CRYAB family genes across different species was observed to be relatively modest. Through genome-wide screening of 22 species representing six insect orders, a total of 235 CRYAB proteins were identified, with *P. puparum* harboring eight CRYAB proteins, indicative of a moderate abundance compared to other species. Intriguingly, evolutionary analysis highlighted *PpCRYAB-4* with potentially intricate differentiation in comparison to other members of the CRYAB family. Furthermore, RNA interference (RNAi) results demonstrated significant regulatory effects on adult lifespan under heat stress at 35°C for *PpCRYAB-4* and *PpCRYAB-5*. These findings lay a groundwork for future investigations into stress resistance mechanisms in parasitic wasps, providing fresh insights for the study of insect resilience amidst the backdrop of global climate change.

## 1 Introduction

One of the most common cellular responses of insects to high temperatures is the induction of heat shock proteins (HSPs) (Jacob et al., 2017). Heat shock proteins were first discovered by the observation of variations in the puffing patterns of chromosomes induced by temperature shock in the fly *Drosophila busckii* (Ritossa, 1962; De Maio et al., 2012). They have been reported as a highly conserved and ubiquitous class of stress proteins or molecular chaperones that are produced in response to various types of stressors (King and MacRae, 2015). Based on their molecular weight, HSPs can be classified into HSP110, HSP90, HSP70, HSP60, HSP40, HSP20, HSP10 *etc.* These HSPs interact and cross-react, forming a complex network (King and MacRae, 2015). Some HSPs, including HSP110, HSP70, HSP60, and HSP10, function in ATP-dependent co-translational or post-translational protein folding (Vos et al., 2008). HSP40 modulates the activity of HSP70, acting as a co-chaperone.

Small heat shock proteins (sHSPs) are a class of molecular chaperones that are the most widespread but also the most poorly conserved (Haslbeck et al., 2005). They are characterized by their relatively small molecular weight, typically ranging from 12 to 43 kDa. The human genome encodes ten members of the sHsp family, including HSPB1 (HSP27), HSPB2, HSPB3, HSPB4 (αA-crystallin), HSPB5 (αB-crystallin or CRYAB), HSPB6 (HSP20), HSPB7, HSPB8, HSPB9, and HSPB10 (Kappé et al., 2003). These sHSP subfamilies share similarities in their structure and functional characteristics, such as low molecular mass, a conserved α-crystallin domain (ACD), the capacity to form large oligomers, a dynamic quaternary structure and induction by stress conditions (Clark et al., 2011). Unlike other heat shock proteins, these sHSPs exhibit chaperone-like activity independent of ATP, functioning as the initial barrier against stress-induced protein aggregation in both animals and humans (Haslbeck et al., 2005; King and MacRae, 2015). These stress-induced proteins have been implicated in many physiological processes, including cellular stress resistance, cytoskeleton stabilization, apoptosis inhibition, membrane fluidity, cellular longevity and resistance to various diseases (Quan et al., 2018). The physiological and pathological functions of sHSPs are commonly believed to be underpinned by their *in vitro* chaperone activity, which involves the prevention of protein aggregation and misfolding that can be detrimental to cells (Fu et al., 2021). For instance, in *Drosophila melanogaster*, the HSP67Bc protein is involved in the recovery of flies from a comatose state under cold stress. It interacts with HSP23 during the recovery phase of flies, contributing to the cold stress response (Malkeyeva et al., 2020). Another example would be the 12 kDa sHSP (HSP12s) subfamily members in the roundworm (*Caenorhabditis elegans*), including HSP12.1, HSP12.2, HSP12.3, and HSP12.6, demonstrated that these HSP12s seem to play crucial physiological roles in suppressing dauer formation and promoting both longevity and reproduction, but hardly exhibiting any chaperone activity (Fu et al., 2021).

*Pteromalus puparum* (Hymenoptera: Pteromalidae) is a cosmopolitan parasitoid wasp that uses a wide range host, and its predominant host to be the pupal stage of *Pieris rapae* (Lepidoptera: Pieridae) (Wang et al., 2008; Wang et al., 2012). *Pteromalus puparum* occurs in 9–13 generations annually in East China, with a high parasitism rate up to 90% in field survey (Hu, 1983). Previous studies documented that the developmental rate, female sex ratio and offspring wasp density per host pupa of *P. puparum* can be significantly influenced by environmental temperature, with the offspring wasp density falling rapidly when the environmental temperature is greater than 30°C (Wang et al., 2008). Six HSP genes were induced at 36°C, including *HSP20*, *HSP40*, *HSP60*, *HSP70*, *HSC70*, and *HSP90* (Wang et al., 2012).

The previous researches about αB-crystallins lacked the explicit identification of the genes in insects, and their functional roles in response to heat stress. Thus, we want to ascertain the evolutionary relationship among the genes of *P. puparum*, and provides direct insight into functional roles of the genes exhibited upon heat stress and their significant influence on wasp lifespan. In this study, we identified the genes belonging to the HSP family in the genome of the parasitoid wasp (*P. puparum*) and investigated their expression patterns under heat stress. We elucidated the evolutionary relationships of CRYAB genes across 22 insect species. Additionally, we validated the regulatory roles of several CRYAB genes in response to heat stress.

## 2 Methods and materials

### 2.1 Insect rearing

The colony of *P. puparum* was continuously cultured in the laboratory for more than ten generations after initial collection in the field in Hangzhou, China (Ye et al., 2020). *Pteromalus puparum* were reared in an artificial climate incubator under 25°C ± 1°C, 60% ± 5% relative humidity and a photoperiod of 16L:8D. After eclosion, adult wasps were mated for 3 days, then one female wasp and one small white butterfly (*P. rapae*) pupa were placed in a cylindrical tube for 24 h to breed wasps.

### 2.2 Identification of HSPs and expression patterns in *Pteromalus puparum*

Heat Shock Protein sequences of *D. melanogaster* and the genome sequences of other 20 insects were retrieved from NCBI GenBank (https://www.ncbi.nlm.nih.gov/genome). We extracted HSP family genes based on the annotated *P. puparum* genome (Ye et al., 2020). The genome sequences were interrogated using BLASTP to acquire homologous candidate protein sequences with an e-value of 10^−5^. All HSP candidate genes were analyzed by HMMER with the Pfam database to ensure that each sequence contained signature domain structures (Prakash et al., 2017). Then the candidate sequences with domain signatures were submitted to BlastKOALA for validation (Kanehisa et al., 2016). The genes ultimately involved were listed in the supplementary material (Supplementary Table S1). We retrieved the expression patterns of HSP family genes from RNA-Seq data, which were uploaded to the NCBI Short Read Archive under the submission number SUB12336765. Two groups of insects were reared at different temperatures (25°C and 35°C) to induce heat stress. Samples were taken at multiple time points, at third hour, sixth hour, 12th hour, day 5, day 10 and day 15. Gene expression levels were quantified using RSEM and analyzed for differential expression using DESeq2 (Li and Dewey, 2011; Love et al., 2014). Differential expressed genes (DEGs) were identified based on *q*-value and fold change criteria that *p*-value <0.05 and the absolute value of fold change greater than 1. *Q*-value is the positive false discovery rate analogue of the *p*-value, and approach to detecting differentially expressed genes (Storey and Tibshirani, 2003). Comparisons were made between treatment and control groups at each time point.

### 2.3 Phylogenetic analysis

Heat Shock Protein family genes were analyzed by MAFFT v7.123b for multiple sequence alignment (Katoh and Standley, 2013). Phylogenetic evolutionary trees were constructed using the maximum likelihood estimation using IQ-tree (Nguyen et al., 2015). The best substitution model was determined by the ModelFinder implemented in IQ-TREE v2.2.0 according to Bayesian Information Criterion (BIC). Models suggested by ModelFinder are: LG + G4 model. Phylogenetic trees were constructed by IQ-TREE with 1,000 ultralfast bootstrap replicates. iTOL was used to visualize the evolutionary trees (Letunic and Bork, 2021).

### 2.4 RNA interference and determination of wasp lifespan

The synthesis of double-stranded RNA (dsRNA) was carried out utilizing a MEGAscript^®^ T7 Transcription Kit (Ambion, Austin, TX). Primers were designed utilizing Primer3web (version 4.1.0), and their corresponding sequences are presented in Supplementary Table S2. At least two pairs of specific primers were created for each evaluated gene. As a negative control, a dsRNA sequence targeting *Luciferase* was generated using the same methodology. The concentration and purity of dsRNA were evaluated using a NanoDrop (Thermo Scientific) spectrophotometer. RNA interference experiments were conducted by injecting dsRNA into the wasps, with dsLuciferase (dsLuc) serving as the control group. Single injections of dsPpCRYAB-1∼5, as well as the mixture of dsPpCRYABs, were performed as six treatment groups. Each dsRNA had a concentration of 3,000 ng/μL, and a volume of 32.2 nL was injected into each wasp. The mixture refers to an equal volume combination of dsPpCRYAB-1∼5, resulting in a concentration of 3,000 ng/μL and a volume of 32.2 nL injected into each wasp. Microinjection was performed using a Nanoject III injector (Model #3-000-207, Drummond Scientific Company, Broomall, PA). The efficiency of RNA interference (RNAi) was assessed using real-time quantitative PCR (RT-qPCR). The lifespans of injected wasps were documented.

Injected adult wasps were collected immediately after eclosion and fed on a 10% sucrose solution, using a modified capillary feeding method (Ja et al., 2007). Sets of ten female wasps were randomly divided into groups as one biological replicate, and each treatment or control group had three biological replicates. Wasp survival was assessed daily to record life spans. The lifespan parameters were analyzed using IBM SPSS Statistics for Mac (Version 25.0; https://www.ibm.com/products/spss-statistics). First, the data were tested for normality using the Shapiro-Wilk test and homogenous of variance using Levene’s test. Then student’s *t* tests were performed. Survival curves were plotted using the Kaplan-Meier method and differences in lifespan between groups were evaluated by the Log-rank (Mantel-Cox) test, using GraphPad Prism version 9.0.0 for Mac (GraphPad Software, San Diego, California, United States, www.graphpad.com).

### 2.5 qPCR analysis

During the lifespan experiments, we carried out parallel sampling of wasps for qPCR analysis. The PrimeScript™ One Step RT-PCR Kit (Takara, Japan) was utilized to synthesize cDNA. For qPCR reactions, ChamQTM SYBR qPCRMaster Mix (Without ROX) (Vazyme Biotech Co., Ltd.) was employed. The qPCR reaction volume was 25 µL with 10 ng cDNA as the template. Each sample was subjected to three biologically independent replicates. Initially, we ensured the specificity of each primer pair. At the end of each qPCR reaction, we included a melt curve ranging from 60°C to 95°C. We performed qPCR for templates with serial dilutions ranging from 10 to 100,000, respectively, to determine the efficiency of the primers and calculate their efficiency values. We selected appropriate primers for gene expression profile determinations based on the specificity and efficiency verifications. 18S ribosome RNA (18S) was used as the reference gene. To calculate relative mRNA expression levels, we used the 2^−ΔΔCt^ method (Livak and Schmittgen, 2001). We plotted the obtained data using GraphPad Prism6 for Mac, where the relative expression levels of genes were presented as means ± SEM. The statistical analysis was performed using two-way ANOVA, and differences were considered significant when *p* < 0.05.

## 3 Results and discussion

### 3.1 Categories of HSPs in the *Pteromalus puparum* genome and their response to heat stress

**FIGURE 1:**
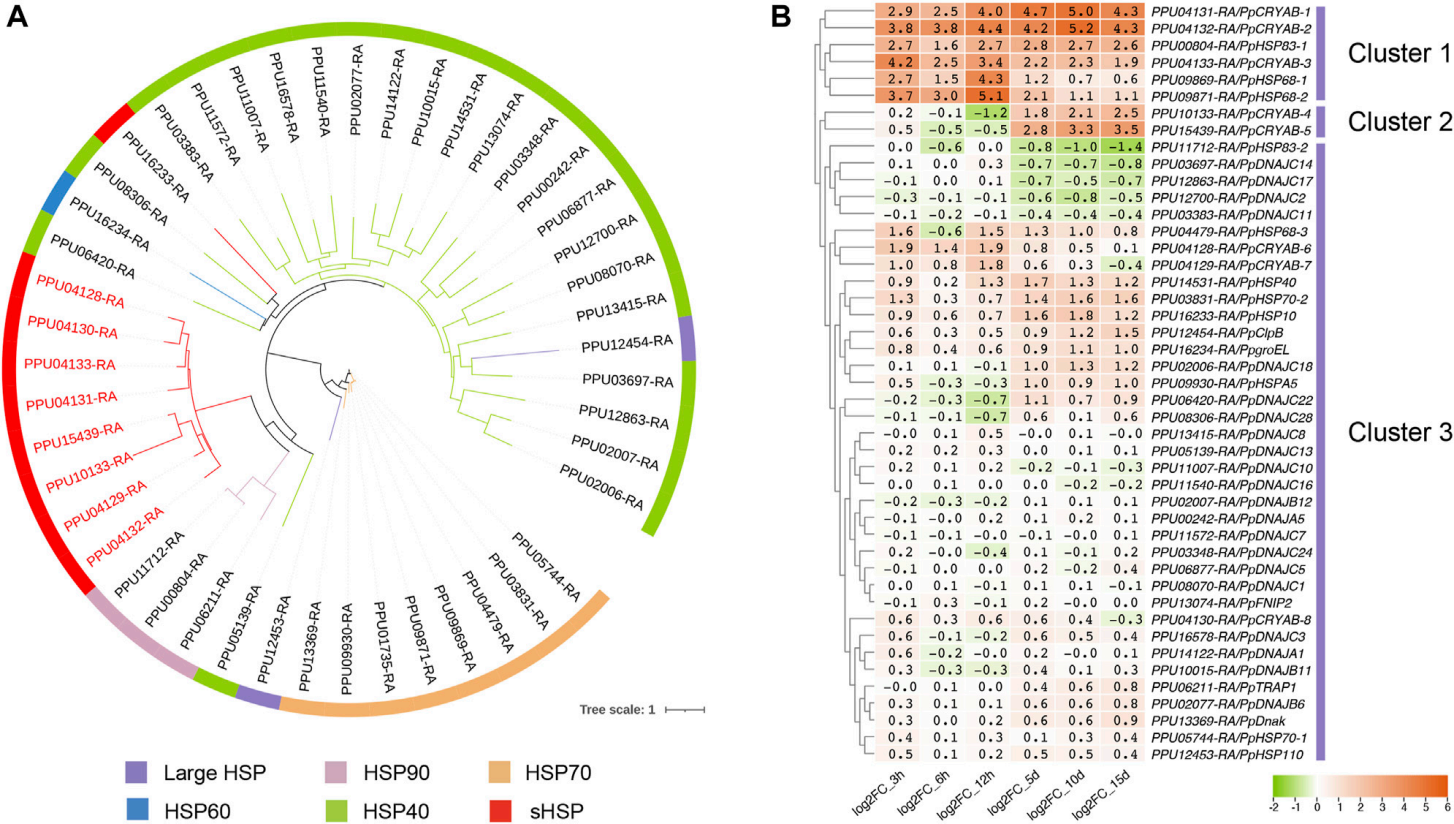
Identification and expression patterns of HSP gene families in *P. puparum* genome. **(A)** Phylogenetic analysis of HSP proteins identified in *P. puparum* genome. The maximum-likelihood tree was constructed using IQ-TREE software with 1,000 ultrafast bootstrap replicates. The CRYABs were indicated by red labels. The identifications, descriptions and molecular mass of HSP genes were listed in Table 1. **(B)** Expression patterns of HSP genes in *P. puparum* genome. Each column represented a time point. Each row represented an HSP gene, which were divided into Cluster 1, Cluster 2 and Cluster 3 according to the clustering analysis. The log2FoldChange value of each gene at a certain age was showed in each cell. Green indicated negative correlation (∼-2) and orange indicated positive correlation (∼+6).

A total of 46 HSPs were identified in the *P. puparum* genome, including two large HSP, three HSP90, eight HSP70, one HSP60, 23 HSP40, and nine sHSPs. Each member possesses a conserved domain corresponding to their respective subfamily (Table 1). HSP40 exhibited the highest gene count among others, with molecular masses ranging from 16.26 to 253.67 kDa. Phylogenetic analysis of *P. puparum* HSPs highlighted a gene, *PPU12454-RA*, identified as ClpB, which clustered together with HSP40 proteins (Figure 1A). ClpB belongs to HSP100 subfamily, involving in ATP-dependent proteolysis (Schlee et al., 2001). The HSP40 family, known as co-chaperons of HSP70 proteins, assists in various protein folding, unfolding and transportation processes (Lubkowska et al., 2021). The phylogenetic analysis suggested a potential functional similarity or interaction between PpClpB and HSP40. This finding consisted with a previous work that reported ClpB interacted with HSP40 (DnaJ) to prevent aggregate formation or to efficiently remove protein aggregates after heat shock (Kedzierska and Matuszewska, 2001). Phylogenetic analysis revealed clustering of HSP10 (encoded by *PPU16233-RA*) and HSP60 (encoded by *PPU16234-RA*) with HSP40. Additionally, we identified nine small heat shock proteins (sHSPs) in *P. puparum* genome, including eight CRYABs and one HSP10. Furthermore, *P. puparum* possesses two subtypes of HSP90, comprising two HSP83 and one TRAP1. TRAP1, also known as HSP75, is a mitochondrial member of the HSP90 chaperone family (Felts et al., 2000).

**TABLE 1:** Identification of HSP gene families in *P. puparum* genome.

Family	Important members	Gene name	Description	Molecular mass (kDa)
HSP40	PPU14531-RA	PpHSP40	DnaJ protein homolog 1	36.62
	PPU14122-RA	PpDNAJA1	DnaJ homolog subfamily A member 1	44.49
	PPU00242-RA	PpDNAJA5	DnaJ homolog subfamily A member 5	75.77
	PPU02077-RA	PpDNAJB6	DnaJ homolog subfamily B member 2	32.79
	PPU10015-RA	PpDNAJB11	DnaJ homolog subfamily B member 11	40.45
	PPU02007-RA	PpDNAJB12	DnaJ homolog subfamily B member 12	42.55
	PPU08070-RA	PpDNAJC1	DnaJ homolog subfamily C member 1	50.16
	PPU12700-RA	PpDNAJC2	DnaJ homolog subfamily C member 2	73.85
	PPU16578-RA	PpDNAJC3	DnaJ homolog subfamily C member 3	55.10
	PPU06877-RA	PpDNAJC5	DnaJ homolog subfamily C member 5	27.41
	PPU11572-RA	PpDNAJC7	DnaJ homolog subfamily C member 7	56.58
	PPU13415-RA	PpDNAJC8	DnaJ homolog subfamily C member 8	30.45
	PPU11007-RA	PpDNAJC10	DnaJ homolog subfamily C member 10	91.58
	PPU03383-RA	PpDNAJC11	DnaJ homolog subfamily C member 11	64.56
	PPU05139-RA	PpDNAJC13	DnaJ homolog subfamily C member 13	253.67
	PPU03697-RA	PpDNAJC14	DnaJ homolog subfamily C member 14	94.03
	PPU11540-RA	PpDNAJC16	DnaJ homolog subfamily C member 16	89.22
	PPU12863-RA	PpDNAJC17	DnaJ homolog subfamily C member 17	37.65
	PPU02006-RA	PpDNAJC18	DnaJ homolog subfamily C member 18	16.26
	PPU06420-RA	PpDNAJC22	DnaJ homolog subfamily C member 22	43.49
	PPU03348-RA	PpDNAJC24	DnaJ homolog subfamily C member 24	16.40
	PPU08306-RA	PpDNAJC28	DnaJ homolog subfamily C member 28	41.67
	PPU13074-RA	PpFNIP2	folliculin-interacting protein 2	153.77
HSP60	PPU16234-RA	PpgroEL	Heat shock protein 60	62.73
HSP70	PPU13369-RA	PpDnak	dnaK, HSPA9; molecular chaperone DnaK	75.17
	PPU09869-RA	PpHSP68-1	Heat shock 70 protein 1/2/6/8	70.63
	PPU09871-RA	PpHSP68-2	Heat shock 70 protein 1/2/6/8	70.41
	PPU04479-RA	PpHSP68-3	Heat shock protein 68	70.94
	PPU05744-RA	PpHSP70-1	Heat shock 70 protein 1/2/6/8	71.29
	PPU03831-RA	PpHSP70-2	Heat shock 70 protein 1/2/6/8	126.97
	PPU09930-RA	PpHSPA5	Heat shock 70 protein 5	72.71
	PPU01735-RA	PpHSP70Ab	Major heat shock 70 protein Ab	12.25
HSP90	PPU00804-RA	PpHSP83-1	Heat shock protein 83	83.36
	PPU11712-RA	PpHSP83-2	Heat shock protein 83	88.13
	PPU06211-RA	PpTRAP1	Heat shock protein 75, mitochondrial	82.14
Large HSP	PPU12453-RA	PpHSP110	Heat shock protein 110	92.88
	PPU12454-RA	PpClpB	ATP-dependent Clp protease ATP-binding subunit ClpB	71.50
sHSP	PPU04131-RA	PpCRYAB-1	Protein lethal(2)essential for life	23.31
	PPU04132-RA	PpCRYAB-2	Protein lethal(2)essential for life	22.74
	PPU04133-RA	PpCRYAB-3	Protein lethal(2)essential for life	22.29
	PPU10133-RA	PpCRYAB-4	Protein lethal(2)essential for life	21.66
	PPU15439-RA	PpCRYAB-5	Protein lethal(2)essential for life	23.04
	PPU04128-RA	PpCRYAB-6	Protein lethal(2)essential for life	28.42
	PPU04129-RA	PpCRYAB-7	Protein lethal(2)essential for life	16.62
	PPU04130-RA	PpCRYAB-8	Protein lethal(2)essential for life	28.65
	PPU16233-RA	PpHSP10	10 heat shock protein, mitochondrial	11.23

To investigate the mRNA expression profiles of HSP genes, RNA-Seq data was obtained from female wasps reared at 35°C and 25°C during the adult stage (Figure 1B). Clustering analysis revealed a prominent response at 35°C for six HSPs (cluster 1), including three CRYABs, two *HSP70*, and one *HSP90*. Notably, *PpCRYAB-1* (*PPU04131-RA*) and *PpCRYAB-2* (*PPU04132-RA*) exhibited the strongest induction throughout the entire adult stage, with upregulation exceeding 36-fold. Two CRYABs, *PpCRYAB-4* (*PPU10133-RA*) and *PpCRYAB-5* (*PPU15439-RA*), displayed a pattern of slightly downregulation followed by significant upregulation (cluster 2), while the remaining HSPs showed no significant changes (cluster 3).

### 3.2 Evolutionary analysis of CRYAB proteins in insects

**FIGURE 2:**
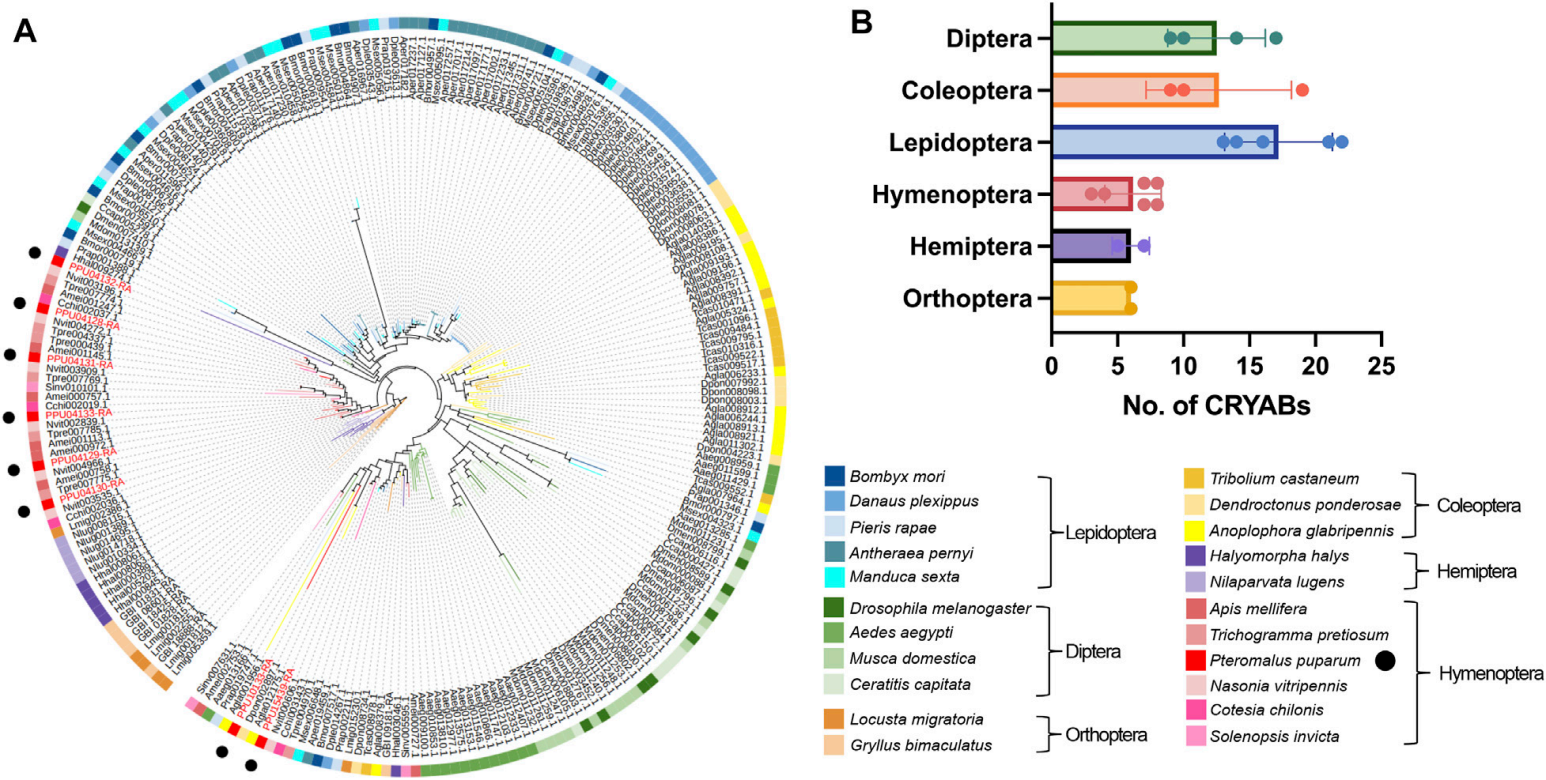
Phylogenetic analysis of CRYAB families in 22 insect species. **(A)** Phylogenetic tree of CRYAB proteins identified in six insect orders. The maximum-likelihood tree was constructed using IQ-TREE software with 1,000 ultrafast bootstrap replicates. The CRYABs of *P. puparum* were indicated by red labels. **(B)** Number of CRYAB proteins identified in six insect orders. Rows represented numbers of CRYAB, and columns represented six orders of insects. The data were denoted as mean ± SEM, and each point represented the amount of CRYAB in each species.

We identified a total of 235 CRYAB proteins from the genomes of 22 insect species, with the presence of the HSP20 domain. To elucidate their evolutionary relationships, we constructed a phylogenetic tree using CRYAB protein sequences from six insect orders and 22 families (Figure 2). The phylogenetic analysis revealed distinct clustering of most CRYAB proteins based on their respective insect orders, with the tree branching out in the following order: Orthoptera, Hemiptera, Hymenoptera, Lepidoptera, Coleoptera, and Diptera (Figure 2A). Notably, among the 10 insect species from Orthoptera, Hemiptera, and Lepidoptera, the number of CRYAB proteins ranged around six. In contrast, the species from Coleoptera, Hymenoptera, and Diptera displayed a higher abundance of CRYAB proteins, with the monarch butterfly (*Danaus plexippus*) having the highest count of 22 CRYAB proteins. Conversely, the host species, the small white butterfly (*P. rapae*), exhibited a relatively lower number of CRYAB proteins, with only 13 identified. Preliminary examination of CRYAB protein counts within each species suggested a tendency for insects belonging to the same order to exhibit similar numbers of CRYAB proteins (Figure 2B). However, it should be noted that this tendency may not be universally consistent and unique. An interesting exception to this trend is observed in the red imported fire ant (*Solenopsis invicta*), which, despite its tolerance to high temperature stress (Xu et al., 2009), was found to have only 3 identified CRYABs.

In the order Hymenoptera, the distribution of CRYABs from the same species was observed to be scattered rather than clustering together, and, interestingly, they tended to cluster with genes from other species. Such a scattered branching pattern suggested that CRYAB genes in Hymenoptera might have undergone complex evolutionary processes, including gene transfer, recombination, or gene duplication events. *Pteromalus puparum* possessed eight CRYAB proteins, while its closed related species, the jewel wasp (*Nasonia vitripennis*), had seven. Notably, seven out of eight CRYAB proteins in *P. puparum* showed high similarity to the seven CRYAB proteins in *N. vitripennis*, as supported by their close proximity on the phylogenetic tree. In contrast, the remaining PpCRYAB-4 appeared distant from other *P. puparum* CRYAB proteins. Furthermore, a subset of 25 CRYAB proteins exhibited significant divergence from other CRYAB proteins within the same species, indicating a high degree of diversity. Notably, this subset included two *P. puparum* CRYAB proteins, that is PpCRYAB-4 and PpCRYAB-5 (Figure 2B). We performed multiple comparisons and constructed evolutionary trees using CRYAB protein sequences from six insects of Hymenoptera, with *D. melanogaster* as the outgroup, and showed that PpCRYAB-4 had the most sequence variation and the longest branches. This suggested that PpCRYAB-4 may have undergone more evolutionary differentiation (Supplementary Figure S1).

### 3.3 Role of PpCRYAB genes in response to heat stress

**FIGURE 3:**
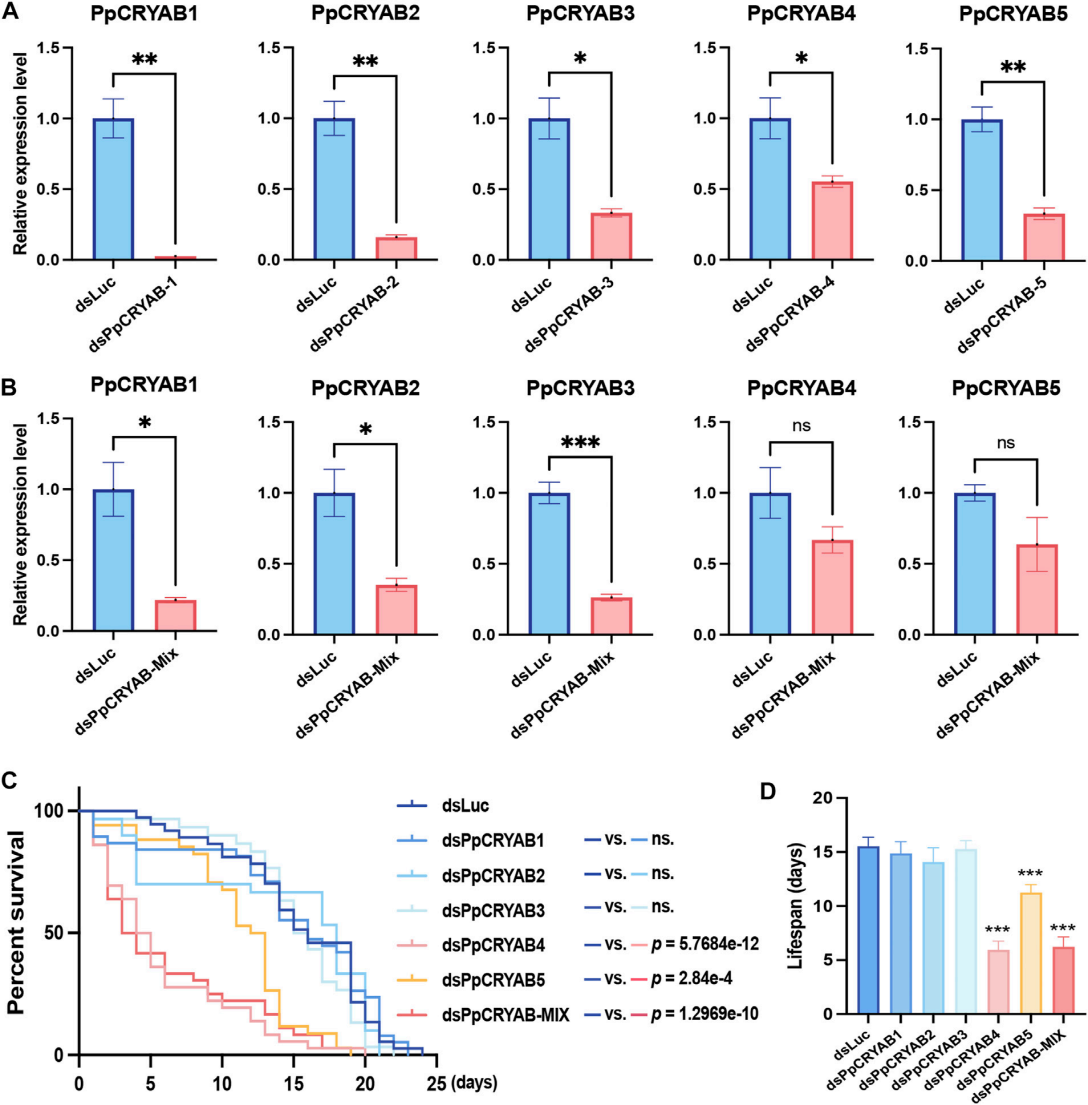
Effects of knock down of *PpCRYAB-1∼5* expressions on the wasps’ lifespan at 35°C. **(A)** Efficiency analyses of each PpCRYAB after single injection of dsPpCRYAB-1∼5. The y-axis showed the relative expression levels of the tested genes. Bar graphs were labeled with * to represent significant differences at *p* < 0.05, ** at *p* < 0.01, *** at *p* < 0.001, ns at *p* > 0.05. The data were denoted as mean ± SEM. **(B)** Efficiency analyses of each PpCRYAB after injection of dsPpCRYAB-Mix. The y-axis showed the relative expression levels of the tested genes. Bar graphs were labeled with * to represent significant differences at *p* < 0.05, ** at *p* < 0.01, *** at *p* < 0.001, ns at *p* > 0.05. The data were denoted as mean ± SEM. **(C)** Survival curve of injected wasps at 35°C. The x-axis represented days, and the y-axis represented the survival rate (%). A t test was performed for each treatment and control group, and the p-values of the results were marked on the right. The ns. represented *p* > 0.05. **(D)** Lifespan of injected wasps at 35°C. The x-axis represented the treatment group, and the y-axis represented mean lifespans of wasps. Bar graphs were labeled with *** to represent significant differences at *p* < 0.001. The data were denoted as mean ± SEM.

Through differential gene expression analysis, five PpCRYAB genes were found to exhibit significant upregulation upon heat stimulation. Based on their expression patterns and evolutionary analysis, our focus narrowed down to *PpCRYAB-1*∼*5*. Knockdown experiments were conducted for *PpCRYAB-1*∼*5*, as well as equal mixture interference using dsRNA targeting these genes. The knockdown efficiency was assessed by measuring the expression levels of the target genes. Both single gene knockdown and injection of a mixture of dsPpCRYABs resulted in decreased expression levels of the target genes (Figures 3A, B). For *PpCRYAB-1∼3*, the decrease in expression levels was significant, with both injection of a single dsPpCRYAB and the mixture dsPpCRYAB-Mix. With injection of a single dsRNA, the reduction of the relative expression level was 97.3% (*p* = 0.0022) for *PpCRYAB-1*, 84.0% (*p* = 0.0024) for *PpCRYAB-2*, 66.7% (*p* = 0.0106) for *PpCRYAB-3* (Figure 3A). With injection of the dsPpCRYAB-Mix, the reduction of the relative expression level was 78.1% (*p* = 0.0151) for *PpCRYAB-1*, 64.8% (*p* = 0.0203) for *PpCRYAB-2*, 73.7% (*p* = 0.0007) for *PpCRYAB-3* (Figure 3B). However, for *PpCRYAB-4* and *PpCRYAB-5*, the expression levels decreased significantly in a single dsPpCRYAB injection, but did not reach significance with the mixture injection. With injection of a single dsRNA, the reduction of the relative expression level was found to be 44.7% (*p* = 0.0406) for *PpCRYAB-4*, and 66.6% (*p* = 0.0022) for *PpCRYAB-5* (Figure 3A). With injection of the dsPpCRYAB-Mix, the reduction of the relative expression level was 33.2% (*p* = 0.1747) for *PpCRYAB-4*, and 36.3% (*p* = 0.1420) for *PpCRYAB-5* (Figure 3B). The lifespan of wasps was analyzed to examine the effects of gene knockdown. In the control group injected with dsLuc, the average lifespan was 15.5 days. Knockdown of *PpCRYAB-1∼3* individually did not cause any significant change in lifespan, with average lifespans of 14.9, 14.1, and 15.3 days, respectively (Figures 3C, D, *p* > 0.05). However, knockdown of *PpCRYAB-4* and *PpCRYAB-5* individually significantly shortened the average lifespan of wasps to 5.9 and 11.3 days, respectively (*p* < 0.05). Injection of the mixture of dsRNA resulted in a 40.0% reduction in the lifespan of the wasps compared to control, decreasing to 6.2 days (*p* < 0.05).

It can be observed that the single knockdown of *PpCRYABs* was more effective in reducing the expression levels. This could be due to the decrease in the mass of each dsRNA component in the mixed injection, leading to a weakened interference effect on each specific PpCRYAB. This indicates that the mixture dsPpCRYAB-Mix did not produce cumulative effects on each PpCRYAB genes. It is worth noting that the result showed a significant reduction in the lifespan of wasps when injected with dsPpCRYAB-Mix, whereas the individual gene knockdown of *PpCRYAB-1∼ 3* did not affect the lifespan. This suggests that simultaneous interference with these three genes affects their protective role in response to heat stress in wasps. It indicates that the functions of *PpCRYAB-1∼3* are redundant and complement to each other during the heat stress process. On the other hand, lifespan experiments revealed a significantly shortened lifespan after the single knockdown of *PpCRYAB-4* and *PpCRYAB-5*. These findings suggest that *PpCRYAB-4* and *PpCRYAB-5* play important roles in heat stress response and the regulation of lifespan in wasps.

The RNAi results indicate that two CRYAB genes play a regulatory role in adult lifespan under heat stress at 35°C, despite all five CRYAB genes showing significant induction in response to heat stress. Notably, *PpCRYAB-4* exhibited a distinct evolutionary trajectory, suggesting potential functional divergence from other CRYAB proteins. Combine with their expression patterns, the two CRYAB genes play a sufficient and necessary role in the response to heat stress. These genes, *PpCRYAB-4* and *PpCRYAB-5*, exhibited both dramatic induction in expression upon heat stress and a significant reduction in wasp lifespan after the knockdown using RNAi.

## 4 Conclusion

In this study, we elucidated the classification, conserved structures, evolutionary relationships, and expression patterns in response to high temperatures of HSP family genes in *P. puparum*. We identified five CRYAB genes that were strongly induced by high temperature, and among them, two CRYAB genes were found to play a sufficient and necessary role in the response to heat stress. These genes, *PpCRYAB-4* and *PpCRYAB-5*, exhibited both dramatic induction in expression upon heat stress and a significant reduction in wasp lifespan after the knockdown using RNAi. Evolutionary analysis highlighted the unique position of *PpCRYAB-4*, suggesting its potential distinct functional characteristics and the need for further investigation. These findings enhance our understanding of the molecular mechanisms underlying stress responses in parasitic wasps and provide potential approaches for developing targeted approaches to enhance insect adaptability in the face of global climate change.

## Data Availability

The original contributions presented in the study are included in the article/Supplementary Material, further inquiries can be directed to the corresponding author.
