# Supplementary material for: Identification and functional analysis of αB-crystallins in Pteromalus puparum
Source: Front Physiol. 2023 Jul 14;14:1214835. doi: 10.3389/fphys.2023.1214835 (PMC10382227; doi:10.3389/fphys.2023.1214835)
Supplement: Supplementary file 1 [file DataSheet1.docx]

Supplementary Material

Identification and functional analysis of CRYABs in *Pteromalus puparum*

Shuxing Lao^1^, Shijiao Xiong^1*^, Qi Fang^1^, Gongyin Ye^1^

^1^State Key Laboratory of Rice Biology and Breeding, Institute of Insect Sciences, Zhejiang University, Hangzhou 310058, China.

# Supplementary Figures


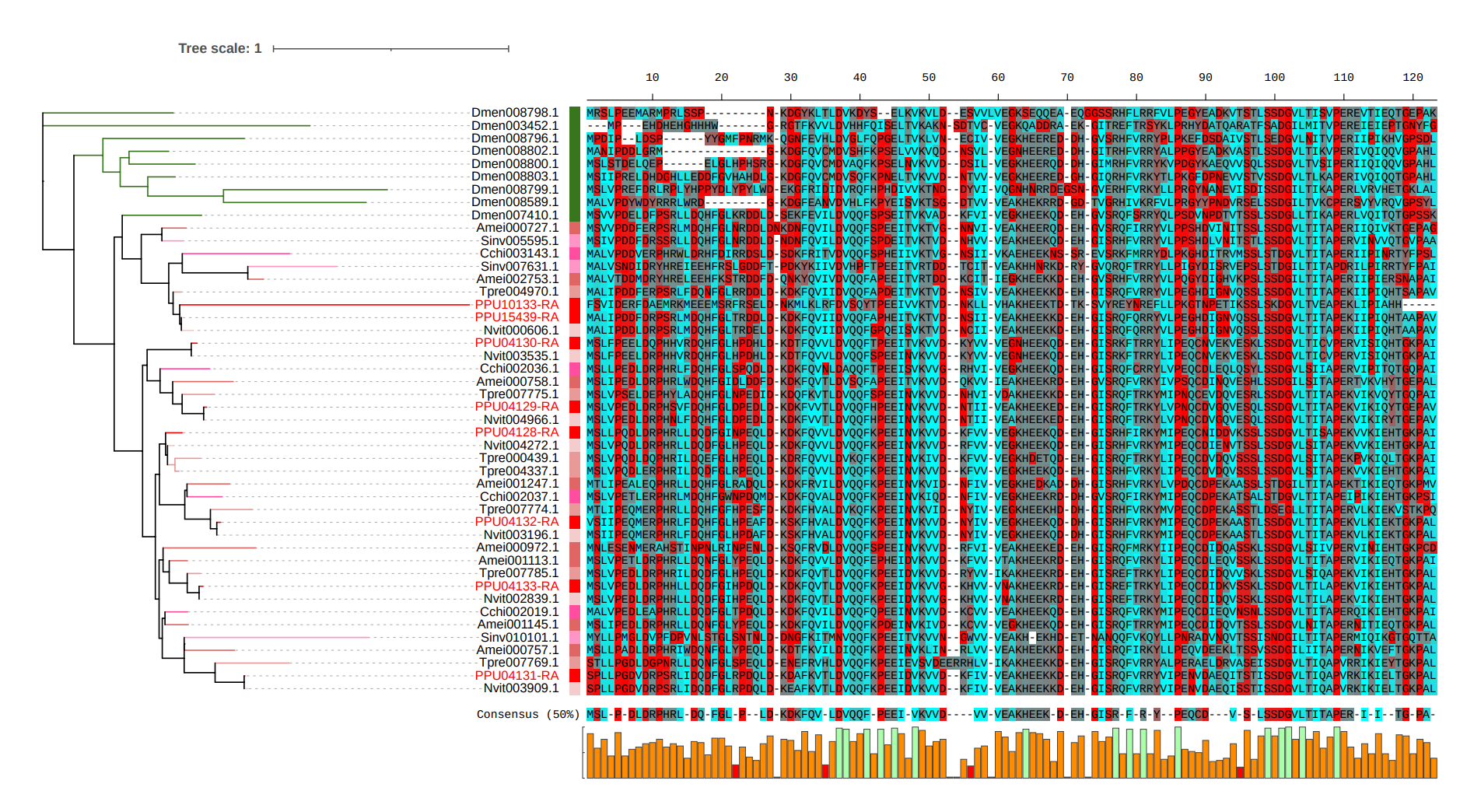


**Supplementary Figure 1.** Phylogenetic analysis and multiple sequence alignment of CRYAB families in *D. melanogaster* and 6 species of Hymenoptera during HS. The maximum-likelihood tree was constructed using IQ-TREE software with 1000 ultrafast bootstrap replicates. The CRYABs of *P. puparum* were indicated by red labels. The multiple sequence alignment was trimmed by trimAl. Fifty percent consensus and analysis was in the bottom right corner.

# Supplementary Tables

**Supplementary Table 1.** CRYAB proteins for evolutionary analysis in Figure 2.

| **Order** | **Species** | | **Protein ID** |  |
| --- | --- | --- | --- | --- |
| Diptera | *Drosophila melanogaster* | | Dmen003452.1 |  |
|  |  |  | Dmen007410.1 |  |
|  |  |  | Dmen008589.1 |  |
|  |  |  | Dmen008796.1 |  |
|  |  |  | Dmen008798.1 |  |
|  |  |  | Dmen008799.1 |  |
|  |  |  | Dmen008800.1 |  |
|  |  |  | Dmen008802.1 |  |
|  |  |  | Dmen008803.1 |  |
|  | *Aedes aegypti* | | Aaeg008959.1 |  |
|  |  |  | Aaeg009100.1 |  |
|  |  |  | Aaeg010853.1 |  |
|  |  |  | Aaeg010866.1 |  |
|  |  |  | Aaeg011429.1 |  |
|  |  |  | Aaeg011546.1 |  |
|  |  |  | Aaeg011599.1 |  |
|  |  |  | Aaeg011747.1 |  |
|  |  |  | Aaeg012103.1 |  |
|  |  |  | Aaeg012333.1 |  |
|  |  |  | Aaeg012407.1 |  |
|  |  |  | Aaeg012977.1 |  |
|  |  |  | Aaeg013153.1 |  |
|  |  |  | Aaeg013166.1 |  |
|  |  |  | Aaeg013285.1 |  |
|  |  |  | Aaeg013575.1 |  |
|  |  |  | Aaeg013810.1 |  |
|  | *Musca domestica* | | Mdom000088.1 |  |
|  |  |  | Mdom011215.1 |  |
|  |  |  | Mdom011223.1 |  |
|  |  |  | Mdom011231.1 |  |
|  |  |  | Mdom011232.1 |  |
|  |  |  | Mdom011240.1 |  |
|  |  |  | Mdom011247.1 |  |
|  |  |  | Mdom011248.1 |  |
|  |  |  | Mdom011253.1 |  |
|  |  |  | Mdom011256.1 |  |
|  |  |  | Mdom011259.1 |  |
|  |  |  | Mdom011261.1 |  |
|  |  |  | Mdom012767.1 |  |
|  |  |  | Mdom013139.1 |  |
|  | *Ceratitis capitata* | | Ccap000427.1 |  |
|  |  |  | Ccap005278.1 |  |
|  |  |  | Ccap006084.1 |  |
|  |  |  | Ccap006087.1 |  |
|  |  |  | Ccap006102.1 |  |
|  |  |  | Ccap006105.1 |  |
|  |  |  | Ccap006116.1 |  |
|  |  |  | Ccap006136.1 |  |
|  |  |  | Ccap006150.1 |  |
|  |  |  | Ccap006172.1 |  |
| Coleoptera | *Tribolium castaneum* | | Tcas001096.1 |  |
|  |  |  | Tcas008978.1 |  |
|  |  |  | Tcas009484.1 |  |
|  |  |  | Tcas009517.1 |  |
|  |  |  | Tcas009522.1 |  |
|  |  |  | Tcas009552.1 |  |
|  |  |  | Tcas009795.1 |  |
|  |  |  | Tcas010316.1 |  |
|  |  |  | Tcas010471.1 |  |
|  | *Dendroctonus ponderosae* | | Dpon002897.1 |  |
|  |  |  | Dpon004223.1 |  |
|  |  |  | Dpon007992.1 |  |
|  |  |  | Dpon008003.1 |  |
|  |  |  | Dpon008063.1 |  |
|  |  |  | Dpon008078.1 |  |
|  |  |  | Dpon008081.1 |  |
|  |  |  | Dpon008098.1 |  |
|  |  |  | Dpon008108.1 |  |
|  |  |  | Dpon008734.1 |  |
|  | *Anoplophora glabripennis* | | Agla001956.1 |  |
|  |  |  | Agla005324.1 |  |
|  |  |  | Agla006233.1 |  |
|  |  |  | Agla006244.1 |  |
|  |  |  | Agla007964.1 |  |
|  |  |  | Agla008379.1 |  |
|  |  |  | Agla008386.1 |  |
|  |  |  | Agla008391.1 |  |
|  |  |  | Agla008392.1 |  |
|  |  |  | Agla008912.1 |  |
|  |  |  | Agla008913.1 |  |
|  |  |  | Agla008921.1 |  |
|  |  |  | Agla009193.1 |  |
|  |  |  | Agla009195.1 |  |
|  |  |  | Agla009196.1 |  |
|  |  |  | Agla009757.1 |  |
|  |  |  | Agla011302.1 |  |
|  |  |  | Agla012175.1 |  |
|  |  |  | Agla014033.1 |  |
| Hemiptera | *Halyomorpha halys* | | Hhal000389.1 |  |
|  |  |  | Hhal000845.1 |  |
|  |  |  | Hhal003046.1 |  |
|  |  |  | Hhal005203.1 |  |
|  |  |  | Hhal008061.1 |  |
|  |  |  | Hhal008063.1 |  |
|  |  |  | Hhal009274.1 |  |
|  | *Nilaparvata lugens* | | Nlug001389.1 |  |
|  |  |  | Nlug008115.1 |  |
|  |  |  | Nlug010334.1 |  |
|  |  |  | Nlug014695.1 |  |
|  |  |  | Nlug014718.1 |  |
| Hymenoptera | *Apis mellifera* | | Amei000727.1 |  |
|  |  |  | Amei000757.1 |  |
|  |  |  | Amei000758.1 |  |
|  |  |  | Amei000972.1 |  |
|  |  |  | Amei001113.1 |  |
|  |  |  | Amei001145.1 |  |
|  |  |  | Amei001247.1 |  |
|  |  |  | Amei002753.1 |  |
|  | *Trichogramma pretiosum* | | Tpre000439.1 |  |
|  |  |  | Tpre004337.1 |  |
|  |  |  | Tpre004970.1 |  |
|  |  |  | Tpre007769.1 |  |
|  |  |  | Tpre007774.1 |  |
|  |  |  | Tpre007775.1 |  |
|  |  |  | Tpre007785.1 |  |
|  | *Pteromalus puparum* | | Ppup010314.1 |  |
|  |  |  | Ppup014232.1 |  |
|  |  |  | Ppup014233.1 |  |
|  |  |  | Ppup014234.1 |  |
|  |  |  | Ppup014235.1 |  |
|  |  |  | Ppup014236.1 |  |
|  |  |  | Ppup014237.1 |  |
|  |  |  | Ppup007683.1 |  |
|  | *Nasonia vitripennis* | | Nvit000606.1 |  |
|  |  |  | Nvit002839.1 |  |
|  |  |  | Nvit003196.1 |  |
|  |  |  | Nvit003535.1 |  |
|  |  |  | Nvit003909.1 |  |
|  |  |  | Nvit004272.1 |  |
|  |  |  | Nvit004966.1 |  |
|  | *Cotesia chilonis* | | Cchi002019.1 |  |
|  |  |  | Cchi002036.1 |  |
|  |  |  | Cchi002037.1 |  |
|  |  |  | Cchi003143.1 |  |
|  | *Solenopsis invicta* | | Sinv005595.1 |  |
|  |  |  | Sinv007631.1 |  |
|  |  |  | Sinv010101.1 |  |
| Lepidoptera | *Bombyx mori* | | Bmor000610.1 |  |
|  |  |  | Bmor000679.1 |  |
|  |  |  | Bmor000719.1 |  |
|  |  |  | Bmor000721.1 |  |
|  |  |  | Bmor000797.1 |  |
|  |  |  | Bmor004721.1 |  |
|  |  |  | Bmor004800.1 |  |
|  |  |  | Bmor004823.1 |  |
|  |  |  | Bmor004828.1 |  |
|  |  |  | Bmor004864.1 |  |
|  |  |  | Bmor004907.1 |  |
|  |  |  | Bmor004957.1 |  |
|  |  |  | Bmor007517.1 |  |
|  |  |  | Bmor007597.1 |  |
|  | *Danaus plexippus* | | Dple003480.1 |  |
|  |  |  | Dple003498.1 |  |
|  |  |  | Dple003530.1 |  |
|  |  |  | Dple003543.1 |  |
|  |  |  | Dple003549.1 |  |
|  |  |  | Dple003553.1 |  |
|  |  |  | Dple003574.1 |  |
|  |  |  | Dple003596.1 |  |
|  |  |  | Dple003608.1 |  |
|  |  |  | Dple003613.1 |  |
|  |  |  | Dple003638.1 |  |
|  |  |  | Dple003652.1 |  |
|  |  |  | Dple003664.1 |  |
|  |  |  | Dple003715.1 |  |
|  |  |  | Dple003756.1 |  |
|  |  |  | Dple003769.1 |  |
|  |  |  | Dple003792.1 |  |
|  |  |  | Dple003807.1 |  |
|  |  |  | Dple003855.1 |  |
|  |  |  | Dple008124.1 |  |
|  |  |  | Dple008166.1 |  |
|  |  |  | Dple014267.1 |  |
|  | *Pieris rapae* | | Prap000954.1 |  |
|  |  |  | Prap001127.1 |  |
|  |  |  | Prap001346.1 |  |
|  |  |  | Prap001388.1 |  |
|  |  |  | Prap001407.1 |  |
|  |  |  | Prap002211.1 |  |
|  |  |  | Prap011476.1 |  |
|  |  |  | Prap011519.1 |  |
|  |  |  | Prap011536.1 |  |
|  |  |  | Prap019715.1 |  |
|  |  |  | Prap019747.1 |  |
|  |  |  | Prap019836.1 |  |
|  |  |  | Prap019872.1 |  |
|  | *Antheraea pernyi* | | Aper000741.1 |  |
|  |  |  | Aper011401.1 |  |
|  |  |  | Aper011596.1 |  |
|  |  |  | Aper016967.1 |  |
|  |  |  | Aper017002.1 |  |
|  |  |  | Aper017017.1 |  |
|  |  |  | Aper017033.1 |  |
|  |  |  | Aper017097.1 |  |
|  |  |  | Aper017127.1 |  |
|  |  |  | Aper017140.1 |  |
|  |  |  | Aper017177.1 |  |
|  |  |  | Aper017182.1 |  |
|  |  |  | Aper017214.1 |  |
|  |  |  | Aper017230.1 |  |
|  |  |  | Aper017233.1 |  |
|  |  |  | Aper017237.1 |  |
|  |  |  | Aper017257.1 |  |
|  |  |  | Aper017296.1 |  |
|  |  |  | Aper017311.1 |  |
|  |  |  | Aper017345.1 |  |
|  |  |  | Aper019459.1 |  |
|  | *Manduca sexta* | | Msex004291.1 |  |
|  |  |  | Msex004323.1 |  |
|  |  |  | Msex004466.1 |  |
|  |  |  | Msex004584.1 |  |
|  |  |  | Msex004616.1 |  |
|  |  |  | Msex004621.1 |  |
|  |  |  | Msex005013.1 |  |
|  |  |  | Msex005025.1 |  |
|  |  |  | Msex005056.1 |  |
|  |  |  | Msex005076.1 |  |
|  |  |  | Msex005095.1 |  |
|  |  |  | Msex005104.1 |  |
|  |  |  | Msex006510.1 |  |
|  |  |  | Msex006648.1 |  |
|  |  |  | Msex007018.1 |  |
|  |  |  | Msex010458.1 |  |
| Orthoptera | *Locusta migratoria* | | Lmig001812.1 |  |
|  |  |  | Lmig001815.1 |  |
|  |  |  | Lmig002255.1 |  |
|  |  |  | Lmig002386.1 |  |
|  |  |  | Lmig005359.1 |  |
|  |  |  | Lmig015230.1 |  |
|  | *Gryllus bimaculatus* | | GBI_01828-RA |  |
|  |  |  | GBI_01831-RA |  |
|  |  |  | GBI_08601-RA |  |
|  |  |  | GBI_09181-RA |  |
|  |  |  | GBI_18425-RA |  |
|  | |  | GBI_18888-RA | |

**Supplementary Table 2.** Primers used in this study.

| **Gene name** | **Primers** |
| --- | --- |
| For dsRNA synthesis | |
| rnaiPpCRYAB2-F | TAATACGACTCACTATAGGGAGATTTTGGACTGGGACTTCACC |
| rnaiPpCRYAB2-R | TAATACGACTCACTATAGGGAGATGACTTTGGGCCATTTTCTT |
| rnaiPpCRYAB3-F | TAATACGACTCACTATAGGGAGATCCACTGCTTTTCTCCGACT |
| rnaiPpCRYAB3-R | TAATACGACTCACTATAGGGAGACACTCCGTCCGAAGACAGTT |
| rnaiPpCRYAB4-F | TAATACGACTCACTATAGGGAGAGACTTCAGCGTCATCGACAG |
| rnaiPpCRYAB4-R | TAATACGACTCACTATAGGGAGATCTTTGCTGAGGGACGACTT |
| rnaiPpCRYAB5-F | TAATACGACTCACTATAGGGAGAAGCAACTCAGCGCAAGTACA |
| rnaiPpCRYAB5-R | TAATACGACTCACTATAGGGAGAAGCACACCGTCAGAGGAGAG |
| rnaiPpcryab1-F1 | TAATACGACTCACTATAGGGAGAACTTTGGCTTGGGATTACGC |
| rnaiPpcryab1-R1 | TAATACGACTCACTATAGGGAGACCCGTCAGCTCGATTTTGAT |
| For RT-qPCR | |
| QrnaiCryab1-F1 | CCCAAAGAGGACAAACAGGA |
| QrnaiCryab1-R1 | CTAGTTCTTCGCGGTCGTC |
| QrnaiCryab2-F1 | ATCTATCATCCCCAAGTTGGTC |
| QrnaiCryab2-R1 | CTGATCGAAGAGCCGATGTG |
| QrnaiCryab3-F1 | CGAGAAAAGACCAGCCAAAA |
| QrnaiCryab3-R1 | CTTTGCCTTCAATGCTGGTT |
| QrnaiCryab4-F1 | CGGTGTCCTGACTGTAGAGG |
| QrnaiCryab4-R1 | GATGGGGATGAGCTTCTCG |
| QrnaiCryab5-F1 | ACGGTGTGCTCACCATCA |
| QrnaiCryab5-R1 | GGTGTGCTGAATGGGAATG |
